# Charge transport in organic nanocrystal diodes based on rolled-up robust nanomembrane contacts

**DOI:** 10.3762/bjnano.8.129

**Published:** 2017-06-19

**Authors:** Vineeth Kumar Bandari, Lakshmi Varadharajan, Longqian Xu, Abdur Rehman Jalil, Mirunalini Devarajulu, Pablo F Siles, Feng Zhu, Oliver G Schmidt

**Affiliations:** 1 Material Systems for Nanoelectronics, TU Chemnitz, Reichenhainer Str. 70, 09107 Chemnitz, Germany; 2 Institute for Integrative Nanosciences, IFW Dresden, Helmholtz Str. 20, 01069 Dresden, Germany

**Keywords:** charge transport, nanomembrane, organic diode, organic nanocrystal, rolled-up nanotechnology

## Abstract

The investigation of charge transport in organic nanocrystals is essential to understand nanoscale physical properties of organic systems and the development of novel organic nanodevices. In this work, we fabricate organic nanocrystal diodes contacted by rolled-up robust nanomembranes. The organic nanocrystals consist of vanadyl phthalocyanine and copper hexadecafluorophthalocyanine heterojunctions. The temperature dependent charge transport through organic nanocrystals was investigated to reveal the transport properties of ohmic and space-charge-limited current under different conditions, for instance, temperature and bias.

## Introduction

Organic semiconductors have been widely applied in developing optoelectronic devices including light-emitting diodes, transistors, sensors [1–3]. Because the large variety of physical properties can be conveniently tuned by manipulating the molecular structure, a clever design of the nanoscale device geometry opens up further intriguing options for novel applications in the fields of optoelectronics and spintronics [4–6]. Organic nanocrystals have drawn much attention in the community because high quality material can be efficiently generated by controlling the nanoscale physical growth and/or chemical synthesis process [7–10]. The deposition of organic conjugated molecules is usually realized by thermal evaporation in vacuum. The molecules have van der Waals interaction with an inert substrate which makes them weakly bonded to the substrate [11]. As a result, by controlling the molecules deposition parameters, such as deposition rate and the substrate temperature, it is possible to obtain either amorphous smooth and continuous organic thin films with thicknesses down to a few monolayers or inhomogeneous organic nanocrystals such as organic nanopillars and nanopyramids [11–13].

However, the fabrication of organic nanodevices based on nanostructures is a persistent challenge due to difficulties in creating non-destructive contacts [14–16]. Although there have been remarkable advances in the methods for vertically contacting a variety of organic thin films, one of the main challenges to perform such fabrication still lies in the preparation of reliable vertical junctions [16]. Mainly due to the inhomogeneous distribution of nanostructures on the substrate surface, the metallic atoms can inter-diffuse and produce unwanted short-circuit junctions during the contact formation [14]. Thus, the fabrication of a reliable robust top contact is expected to solve this tedious problem. Part of the authors of this report have developed a novel ‘rolled-up nanotechnology’ to tackle this challenge [17–19]. By this method, strained nanomembranes are released from a substrate surface and the elastic relaxation of the built-in strain gradient triggers a self-rolling process of the nanomembranes. The strained nanomembranes roll-up into full microtubes and finally land on top of the organic nanostructures, e.g., self-assembled monolayers and organic nanopyramids [17,19]. Compared to other ‘soft’ contact methods developed recently, including chemical binding [20–21], indirect evaporation [22–23], ‘ready-made’ approaches [24–25], and robust mechanical contacts [26–29], the rolled-up nanotechnology provides the precise positioned electrodes and high fabrication yield of array devices, and does not require the chemical modification of functional organic layers. Furthermore, the candidate materials for rolled-up nanomembranes are metals, ferromagnetic layers, oxides, and complex materials, of which the various properties of thin solid films, e.g., work function and magnetic properties, can be utilized to develop novel functional organic devices [30–31]. In our previous report, organic nanocrystal diodes have been successfully developed, in which rolled-up nanomembranes provide robust contacts to fully unleash the advantages of organic nanocrystals for sensing gas molecules [19]. Apart from the demonstration of functional nanodevices, the investigation and understanding of charge transport mechanisms across the organic nanostructure is a key topic nowadays for developing and optimizing novel nanostructured devices [8–9,32–33].

In this work, we fabricate organic nanocrystal diodes sandwiched between flat metal electrode and rolled-up nanomembrane electrode contacts. The nanocrystals consist of vanadyl phthalocyanine (VOPc) and copper hexadecafluorophthalocyanine (F_16_CuPc) heterojunctions. The temperature dependent current–voltage behaviors were investigated to unveil the charge transport properties of the nanocrystals. As most of the well-studied charge transport systems are based on planar or vertical bulky organic thin-film devices [34], the conduction mechanism in this report will provide a helpful insight into the charge transport in nanoscale systems.

## Results and Discussion

**Figure 1:**
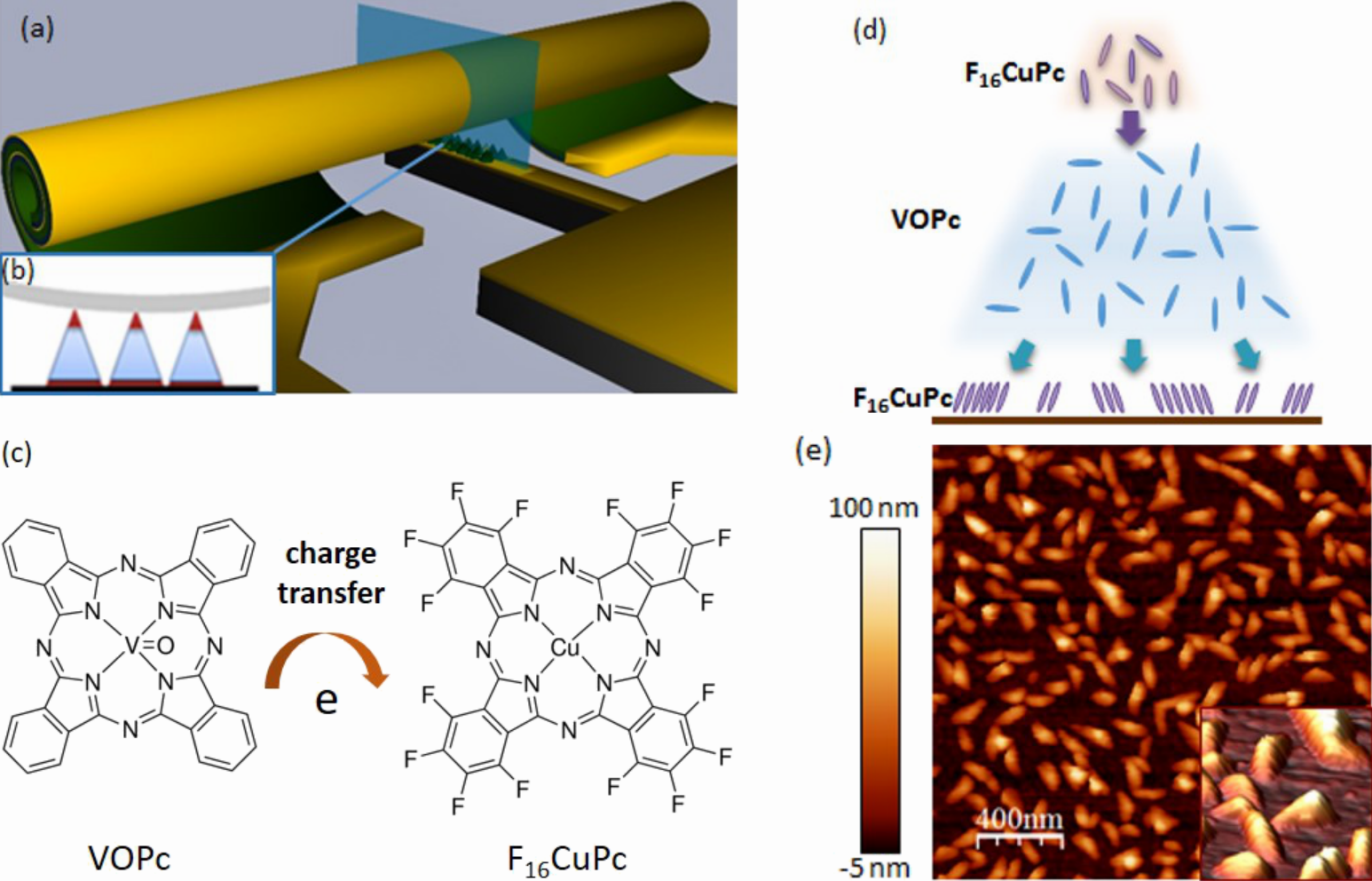
(a) Schematic picture of organic nanocrystal diode with rolled-up contact electrode. (b) Schematic picture of the vertical junction with the nanopyramid sandwiched between the Au mesa electrode and Au tube electrode. (c) Molecular structures of VOPC and F_16_CuPc. (d) Illustration of the F_16_CuPc/VOPc/F_16_CuPc nanopyramid. (e) AFM topography image of the F_16_CuPc/VOPc/F_16_CuPc nanostructures.

The fabrication protocol of the organic nanocrystal diodes is the same as in our previous reports [19]. The fabrication yield of the devices contacted by rolled-up electrodes on the single chip can achieve more than 95% owing to the reliable parallel nanofabrication when the whole process is carefully performed. To study the charge transport properties of the crystalline heterojunction nanopyramids, three kinds of organic nanopyramids were grown on well-defined bottom Au finger electrodes (Au mesa), i.e., pure VOPc (10 nm), F_16_CuPc (1 nm)/VOPc (9 nm) and F_16_CuPc (1 nm)/VOPc (8 nm)/F_16_CuPc (1 nm). The thicknesses are the nominal values detected by the thickness monitor. However, the deposited molecules form inhomogeneous nanopyramids during growth. The heights of the nanopyramids range between 50–100 nm, as observed from atomic force microscopy (AFM) measurements. Figure 1 shows the device configuration and molecular structures. After formation of organic nanopyramids, the rolled-up Au tube electrodes land on top and form reliable contacts. The geometric contacting area between organic and finger electrodes is estimated by considering the circumference of the tube electrode and the width of the mesa electrode, which is on average about 8 μm^2^. An uncertainty concerning the effective electrical contact to the tube electrode remains, because the total number of current pathways across the crystalline nanopyramids is experimentally difficult to determine. The morphology properties of organic layer will determine the effective contact area to a great extent. For instance, a smooth organic layer or self-assembly monolayer has larger contact area when the tube lands on top, while nanopyramid geometry restricts the contact area only within the limited peaks’ surface which can touch the tube. Due to geometry deviation of single nanopyramid structure, it is quite challenging to precisely predict how the single tube surface contacts with nanopyramids and calculate the contact area. As a compromise, in this report the transport properties will be deducted based on the electrical current through device, instead of the intrinsic conductivity of single organic nanostructures.

**Figure 2:**
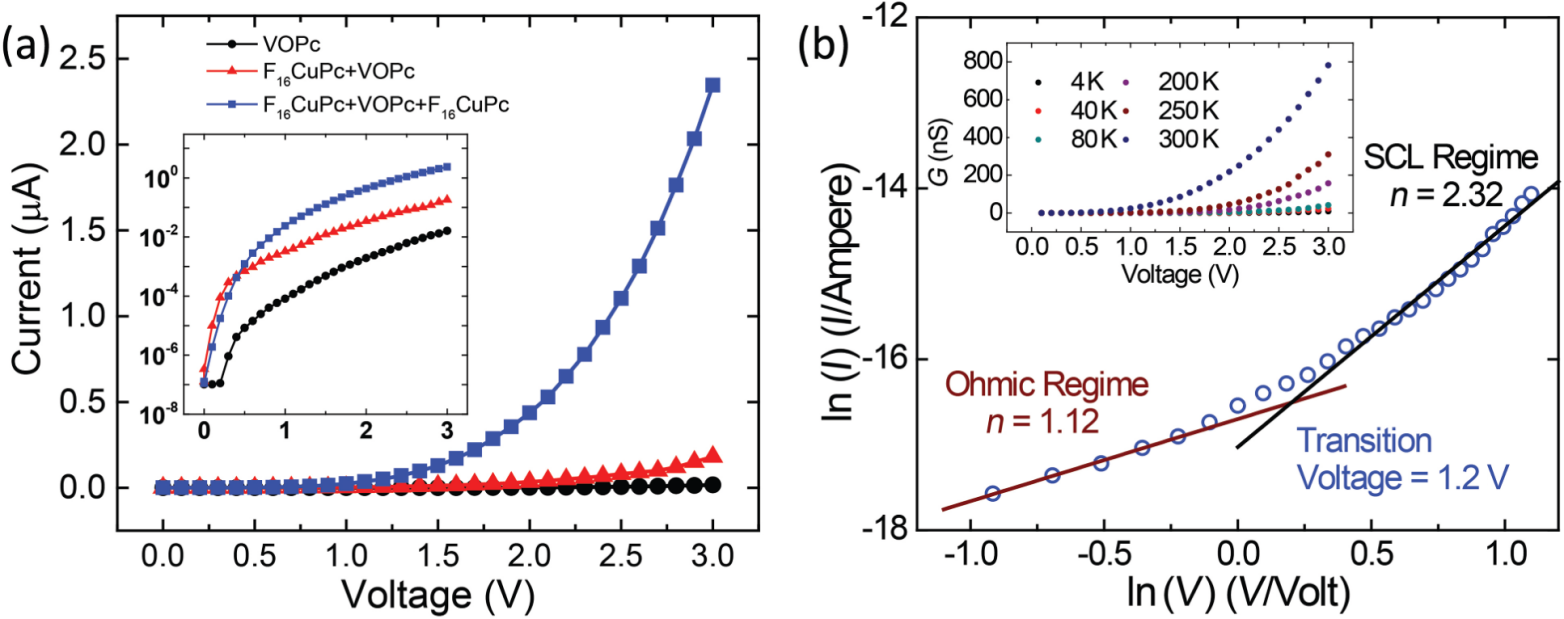
(a) *I*–*V* characteristics of three kinds of nanopyramid structures: pure VOPc (black), F_16_CuPc/VOPc (red) and F_16_CuPc/VOPc/F_16_CuPc (blue), (b) ln(*I*)–ln(*V*) plot showing the transition of transport regimes from ohmic to SCL.

To study the charge transport properties of the crystalline nanopyramids, an electrical characterization is performed by measuring the current–voltage (*I–V*) characteristics. As shown in Figure 2a, the strong charge transfer (CT) between VOPc and F_16_CuPc causes the heterojunction nanopyramids with double F_16_CuPc buffer layers to experience much-improved charge injection/transport under the positive bias, and a smaller open voltage compared to the devices consisting of pure VOPc and single F_16_CuPc buffer layer. As introduced in our previous report [19], the Schottky barrier due to the poor electric contact between the organic material and the electrodes will restrain the charge transport in the diodes consisting of pure VOPc or organic nanostructure with single F_16_CuPc buffer layer. In this report, we will focus on the investigation of charge transport in the F_16_CuPc/VOPc/F_16_CuPc organic nanopyramid diode by applying a forward bias to the tube electrode.

With the F_16_CuPc buffer layers the electrical contacts between the organic nanopyramids and the tube electrode are ohmic at room temperature. A typical plot of the natural logarithm of the current versus natural logarithm of the voltage (ln(*I*)–ln(*V*)) is shown in Figure 2b, which allows us to determine two transport regimes. The curve has a slope of *n* = 1.12 for low bias and a slope of *n* = 2.32 for a high bias. The transition voltage is about 1.2 V. According to the Mott–Gurney Law the transport can be regarded as ohmic in the low bias regime and space-charge-limited (SCL) in the high bias regime [35], which agrees with our previous report [19,36].

**Figure 3:**
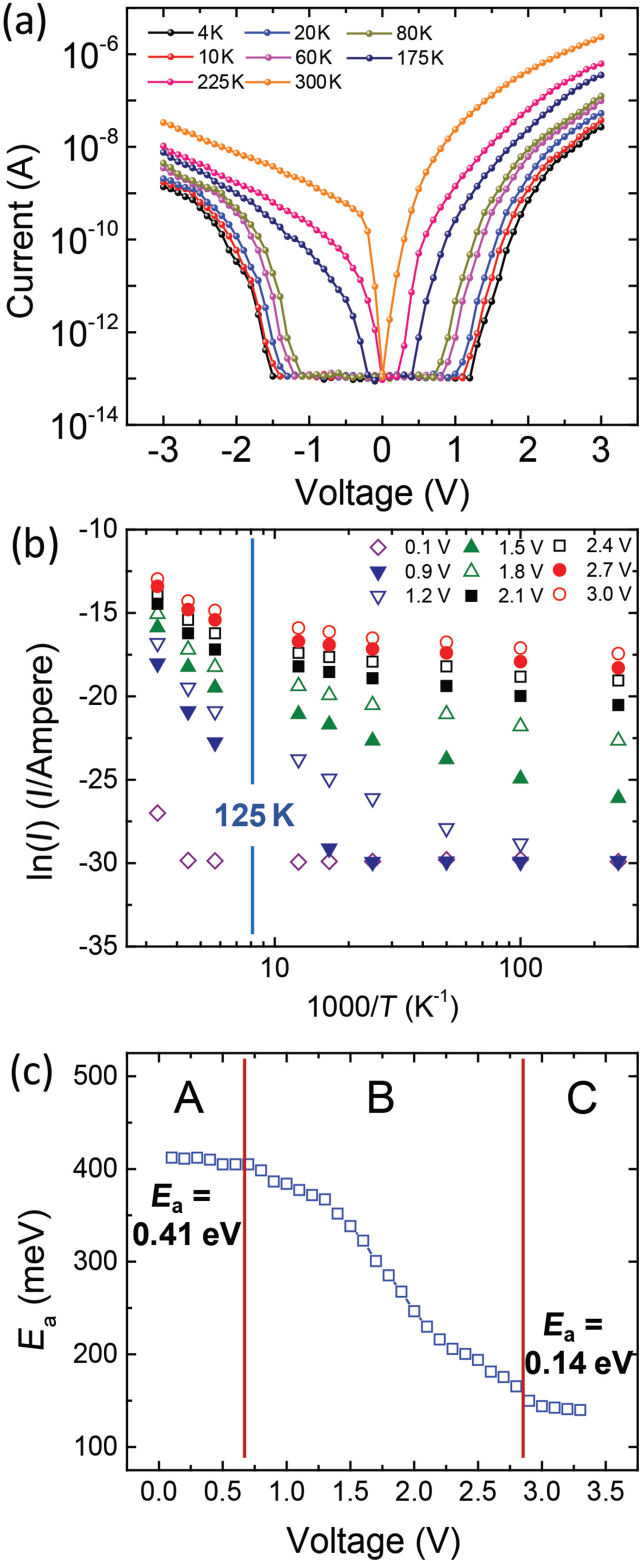
(a) Current–voltage characteristics of Au/F_16_CuPc/VOPc/F_16_CuPc/Au diode as a function of temperature. (b) Current–temperature characteristics at different voltages. (c) Applied voltage dependence of thermal activation energy.

To assess the transport process of the vertical nanopyramid device based on the Au mesa electrode/F_16_CuPc/VOPc/F_16_CuPc/Au tube electrode, *I–V* measurements at different temperatures were performed, as shown in Figure 3a. Similar to the current–voltage characteristics at room temperature, the current under forward bias remains dominant also at lower temperatures. By plotting the temperature dependent current behavior (ln(*I*)–1000/*T*) under different bias we obtain two distinct regions with different slopes the change-over of which occur at around 125 K, as shown in Figure 3b. The curves for *T* > 125 K (left part) show more pronounced temperature dependence, which indicates that thermal activation plays an important role during the transport [37]. Both the left and right regions of Figure 3b are well-described by the classical Arrhenius relation.

[1]
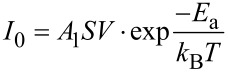


where *E*_a_ is the activation energy, *k*_B_ the Boltzmann’s constant, *A*_1_ the pre-exponential factor, and *S* the estimated contact area.

The occurrence of two thermally activated regions can be explained as the following: for lower temperature, the CT effect between VOPc and F_16_CuPc becomes weak and the contact between the organic material and the electrodes lose their ohmic contact properties. Thus, the current is mainly governed by the Schottky barrier. For higher temperature and higher bias, the current depends on the charge transport ability which is limited by the hopping mobility. For higher temperature and lower bias, the ohmic current is dominated by the temperature dependent carrier density which is provided by the CT between VOPc and F_16_CuPc. In the left part of the curves which are subject to higher activation energy, the activation energy *E_a_* is plotted for each bias voltage.

As shown in Figure 3c, the *E*_a_ as a function of applied voltage *V* can provide more direct information about the thermal activated transport progress above 125 K. The plotted region can be divided into three regimes. Region A under low bias corresponds to the ohmic conduction region. The amount of current is mainly determined by the density of carriers. With low electric field, the injected mobile carriers are much lower than the carriers generated from CT effect between VOPc and F_16_CuPc, therefore, the current is mainly due to the movement of CT mobile carriers, of which the density is subject to thermal activation. The activation energy *E*_a_ is almost constant with decreasing voltage, and calculated to be about 0.41 eV for the voltages below 0.7 V, which is regarded as the CT energy between VOPc and F_16_CuPc. Region C under high bias conditions corresponds to the complete SCL region. As discussed above, the amount of current is mainly determined by the hopping mobility as the amount of injected carriers are much higher than the carriers generated from CT. The activation energy *E*_a_ is almost constant with increasing voltage, and calculated to be about 0.14 eV for the voltages above 2.8 V, which is regarded as the activation energy for the carrier hopping transport [34–35,38]. Region B is the transition region between ohmic and SCL current. Here, the activation energy decreases due to the increase of the charge carrier injection with increasing bias, while the hopping motion in nanocrystals gradually dominates the charge transport. This corresponds to the *I–V* trace in Figure 2b, which shows a smooth transition from ohmic to SCL. As shown in the upper inset of Figure 2b, the voltage dependence of the conductance (*G*) demonstrates that with increasing bias the conductance increases non-linearly and with decreasing temperature the conductance decreases correspondingly. It is worthy to compare here with the diodes consisting of self-assembly monolayer contacted with rolled-up tube electrodes, which is previously reported by some of the authors of this contribution [17]. The transport in such diode is subject to tunneling and field emission mechanisms due to the ultra-thin smooth film, while in the present report the thermal activated transport via bulk nanostructures dominates the transport progress.

## Conclusion

In summary, in this work we investigated the charge transport in F_16_CuPc/VOPc/F_16_CuPc organic nanocrystal diodes, which are contacted by robust metallic rolled-up nanomembranes. The temperature dependent measurement results demonstrate in the temperature region above 125 K carrier injection, and the hopping mechanism dominates the transport through the organic nanocrystals. These conclusions prove that with the assistance of the charge transfer effect, the soft yet robust contacts generated from rolled-up nanotechnology provide an efficient route for fabricating reliable organic nanocrystal electronic and spintronic devices.
